# Knowledge and awareness of emergency department residents and physicians regarding the management of dentofacial traumatic injuries: a cross-sectional study

**DOI:** 10.1038/s41405-024-00267-8

**Published:** 2024-11-09

**Authors:** Tahoora Yousuf, Muhammad Subhan Khan, Robia Ghafoor

**Affiliations:** 1 Operative Dentistry & Endodontics, Department of Surgery, Aga Khan University Hospital, Karachi, Pakistan; 2 Department of Medicine, Aga Khan University Hospital, Karachi, Pakistan

**Keywords:** Health care, Dental trauma

## Abstract

**Background:**

Majority of patients with Dento-Facial Traumatic Injuries (DFTI) seek initial care at an Emergency Department (ED). The timely management of DFTI is of utmost importance in determining long-term prognosis of the tooth and the patient’s overall quality of life. Thus, knowledge and awareness of ED residents & physicians regarding the management of DFTI is crucial for better patient outcomes. Numerous studies have investigated the knowledge of ED specialists regarding initial management of dental trauma, however, scientific evidence in this domain is scarce in our region.

**Objective:**

The aim of this study was to evaluate the knowledge and awareness of ED residents and physicians regarding diagnosis and management of DFTI using a web-based survey on REDCap.

**Methods:**

An observational cross-sectional study was conducted among the ED residents and physicians of private and public hospitals in Karachi, Pakistan. Data was collected through a web-based questionnaire, sent via email to the participants. The survey comprised of questions assessing their knowledge regarding the diagnosis and management of various dental and maxillofacial injuries. Responses from the participants were graded as low, moderate or high knowledge levels according to a pre-determined criteria. Pearson’s chi-square test was applied to determine the association between knowledge scores.

**Results:**

The total response rate was 47.6%. Out of 116 participants, 49 (42%) responses were received from physicians and 67 (58%) from residents. The overall knowledge level of participants was low (46.6%) and there was no significant difference in the knowledge level between residents and physicians (*p* = 0.157). Participants who had received formal training in dental trauma (*p* = 0.038) and those with more years of clinical experience (*p* = 0.004) had higher knowledge scores, that were statistically significant.

**Conclusions:**

The knowledge and awareness of ED residents and physicians in dental trauma management was generally low. Specialized training courses are required to provide timely and adequate management of traumatic dental injuries in order to improve patient-related outcomes.

## Introduction

Dento-Facial Traumatic Injury (DFTI) is one of the most prevalent health issue globally. A systematic analysis by Petti et al. estimates that approximately 900 million individuals worldwide, aged 7–65 years, have sustained traumatic dental injuries to their permanent teeth [1]. These injuries vary in severity ranging from a cracked tooth to more complex ones affecting several teeth, facial bones, and adjacent soft tissues [2, 3]. The management of DFTI in the Emergency Department (ED) requires a multidisciplinary approach for better outcomes; i.e. close coordination between restorative dentists, maxillofacial surgeons, pediatricians, etc [4]. Any delay in treatment may lead to high morbidity, more complicated outcomes, requiring complex interventions in the future [4, 5].

Trauma to the maxillofacial area involves damage to both dental hard and soft tissues, as well as the maxillomandibular region [4]. Dental trauma may be in isolation or compounded with other bodily injuries of varying severity [6]. These injuries may include complicated and uncomplicated crown/root fractures, subluxations, luxations, and avulsion of primary as well as permanent teeth [2, 6]. Traumatized teeth require urgent management and any delay in the provision of treatment adversely affects short and long-term outcomes and the longevity of the teeth [4, 5, 7]. The potential complications of delayed treatment include external/ internal root resorption, ankylosis, and bone loss which ultimately leads to tooth loss [4, 8]. Since the majority of dental injuries affects the anterior maxilla in children or teenagers, these injuries also have a significant negative impact on self-image, psychological well-being, and ultimately the quality of life of patients [9].

The etiology of DFTI includes physical violence, sports injuries, and motor vehicle accidents, amongst others [10]. Consequently, it’s quite common for affected individuals to seek care at an ED before going to a dental practice [5]. Therefore it is imperative that the ED residents and physicians have critical knowledge regarding the diagnosis and management of traumatic dentofacial injuries, adhering to the International Association of Dental Traumatology (IADT) guidelines [6]. Previous studies in South America and Germany have concluded that ED specialists possess insufficient knowledge regarding the awareness and management of dental trauma [11, 12]. Another study by Losier et al. concluded that Canadian ED physicians feel relatively unprepared to treat oral-related problems because their knowledge and skills regarding dental trauma management is inadequate [13]. Moreover, as suggested by the literature, there is scarce evidence regarding the knowledge of the ED physicians and residents for the management of DFTI in our region, hence warranting further investigation.

Therefore, the aim of this cross-sectional study was to evaluate the knowledge, education and awareness of ED residents and physicians regarding the diagnosis and management of DFTI using a web-based survey on REDCap.

## Materials and methods

This study was a cross-sectional survey conducted in accordance with the World Medical Association Declaration of Helsinki (2008) over a period of three months (September 2023–November 2023) [14]. Prior to the commencement of the study, exemption was obtained from the ethical review committee of the institution (ERC exemption #: 2023-9240-26466).

### Sample size

The sample size was calculated using WHO calculator (sample size determination in Health studies, WHO) using the function ‘estimating a population proportion with specified absolute precision’ [15]. In the study by Coskun et al. the percentage of physicians who received trauma training was reported to be 61.7% [4]. Using these data at an absolute precision of 5% and a confidence interval of 95%, the sample size was calculated to be 91 individuals. To account for dropouts, it was then inflated by 25%, yielding a total of 116 participants to answer the study question.

### Inclusion criteria

- Residents and physicians who were working in the ED of private and public hospitals in Karachi, Pakistan.
- Residents from first through fifth years of post-graduate training.

### Exclusion criteria

- Residents and physicians of other specialties rotating in the ED of public and private hospitals

### Questionnaire and content validation index calculation (CVI)

The IADT has published guidelines to assist dentists, health providers, and patients in the management of DFTI [8, 16, 17]. The present survey comprised of a self-administered customized electronic questionnaire, based on questions from former studies and information contained in IADT guidelines using the REDCap software (Supplementary File 1) [4, 6, 8, 16, 17]. The survey comprised of three parts, including questions in both multiple-choice and yes/no formats. The first section included informed consent followed by ten questions regarding the participants’ demographics, professional experience, the presence/absence of a dentist in the family, trauma training, dental trauma training, knowledge of the IADT guidelines, and the presence/absence of a consultant dentist at their institution. The second part consisted of nine questions related to tooth anatomy, differences between primary and permanent teeth, management of dislocated teeth and crown fractures, and management of avulsion in primary and permanent dentitions. Finally, the last section comprised questions regarding the participants’ knowledge of trauma in the oral and maxillofacial region.

Furthermore, the questionnaire was modified according to our study population and setting, and CVI was calculated to ensure its validity & for any uncertainties in the questions. A panel of 6 experts was tasked with reviewing the questionnaire items for relevance and clarity. These 6 experts included a general dentist, dental hygienist, registered nurse, microbiologist, biostatistician, and epidemiologist. Each questionnaire item was assessed by the experts based on relevance and clarity and was rated on a scale of ‘1’ to ‘4’ with ‘1’ being not relevant/not clear to ‘4’ being highly relevant/very clear. A score of ‘1’ or ‘2’ rated by experts is designated as 0 while a score of ‘3’ or ‘4’ is designated as 1. Once all experts have rated the items, CVI is calculated by the sum of the number of experts who rated the item as ‘3’ or ‘4’, divided by the total number of experts. This provides a value of 0.99 confirming the accuracy and validation of the questionnaire.

The criteria by Yigit et al. was used to assess the knowledge levels, graded as low, moderate, or high if the participants responded with <6, 6–10 and >10 correct answers, respectively [6].

### Data collection

The validated questionnaire was emailed to 340 ED residents and physicians registered under College of Physicians & Surgeons Pakistan (CPSP), encompassing the entire population from both private and public hospitals in Karachi, Pakistan. ED residents who are trainees pursuing post-graduation in emergency services, and physicians, who have completed their training in ED and become consultants, were included in the survey. Despite the extensive distribution, only 162 individuals actively took part in the survey. The email addresses were obtained from the homepages of local regulatory authorities and sorted by hospital authorities to reach out to ED employees. One week following the initial invitation, a reminder was sent to all recipients. Participants submitted a data protection declaration and provided their consent before completing the questionnaire anonymously. Only the completed survey forms (*n* = 116) were considered, while incomplete forms (*n* = 46) were excluded from the analysis.

## Statistical analysis

Statistical analysis was performed using SPSS version 26.0. The response rate of the participants was calculated based on the number of responses received divided by total number of surveys sent and presented as percentage. Descriptive statistics was performed and all categorical data (designation of the participant, years of experience, type of health sector, and knowledge level according to grading) was presented as percentages. Pearson’s chi-square test was used to compare and assess the relationship between respondents’ knowledge level and categorical variables. The level of significance was kept at *p* < 0.05.

## Results

**Fig. 1:**
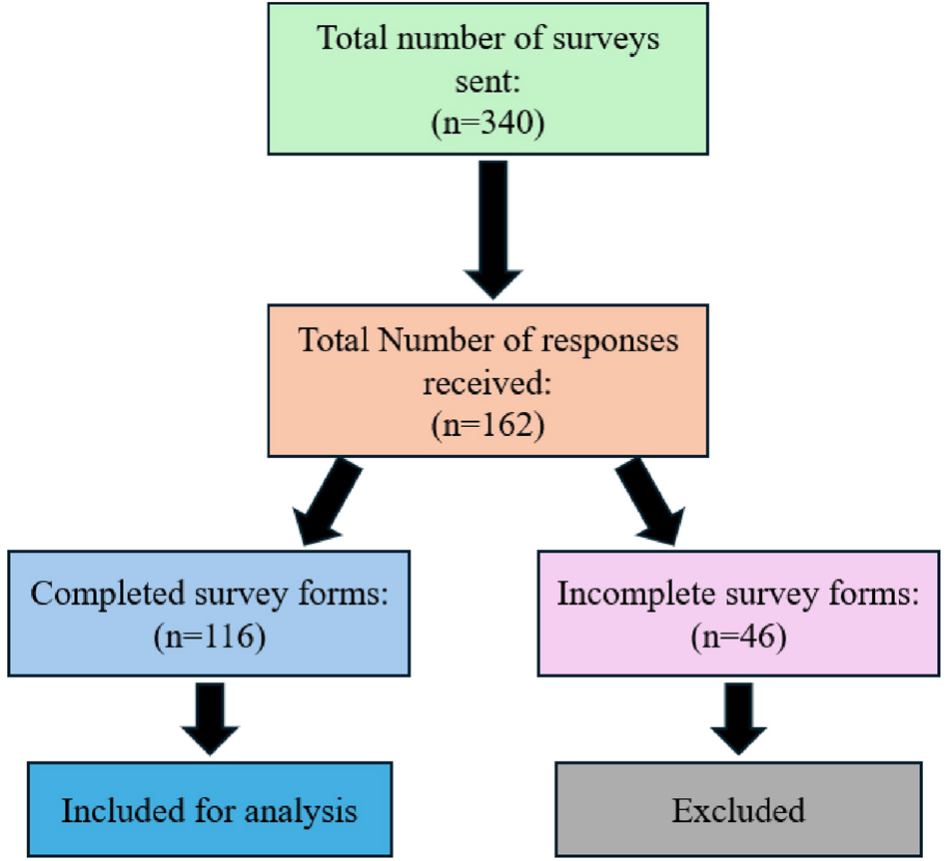
Graphical representation of survey responses. Flow chart illustrating the total number of surveys sent and the responses received.

Out of 340 ED residents and physicians surveyed in Karachi, Pakistan, 162 participants responded yielding a response rate of 47.6%. After further scrutinization, 116 of these were found to be fully completed surveys, meeting the required sample size. These 116 completed surveys were included in the analysis, while the 46 incomplete surveys were excluded (Fig. 1). Of those with completed surveys 42.2% were ED physicians and 57.8% were ED residents. Additionally, 44% of the participants were from public hospitals and 56% from private hospitals. Demographic data, years of experience, awareness of IADT guidelines, formal dental trauma training as well as access to a consultant dentist are shown in Table 1.

**Table 1:**
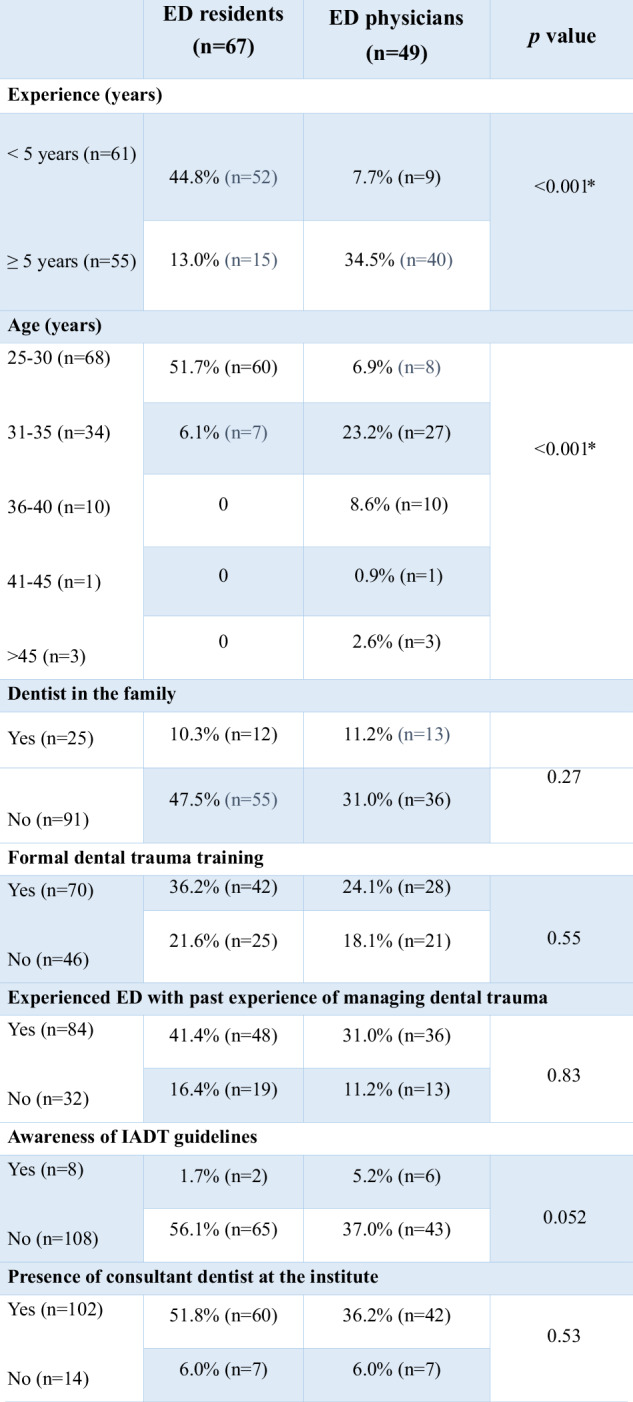
Demographic characteristics and general data of the participants (n = 116).

*ED* emergency department, *IADT* international association of dental traumatology.

*p-value ≤ 0.05.

Among the participants, 60% stated that they have received formal dental trauma training. Overall, 6.90% of the participants knew the IADT trauma protocols. The overall knowledge level of the participants was low (46.6%) followed by moderate levels of knowledge (41.4%). Further sub-stratification analysis revealed a greater number of physicians (18.4%) in the high-level knowledge category compared to residents (7.5%). However, there was no statistically significant difference among the groups in terms of their knowledge levels (*p* = 0.157). Participants with greater years of experience and formal dental trauma training showed a statistically significant difference in the knowledge level (*p* < 0.05). Factors affecting the knowledge levels of participants are shown in Table 2.

**Table 2:**
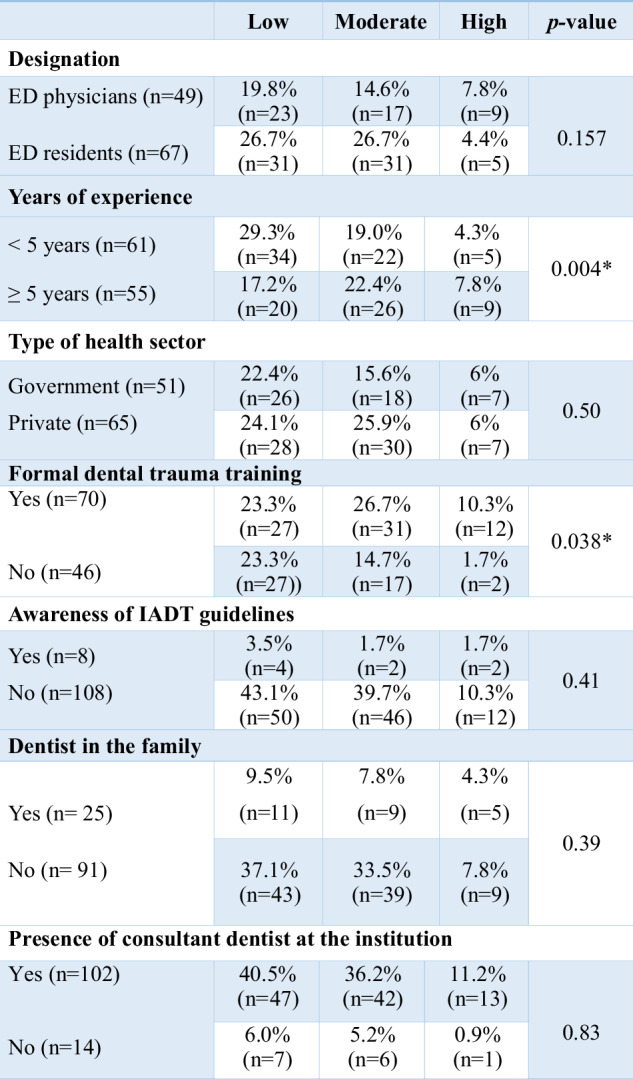
Analysis of factors affecting knowledge level.

Peason chi-square test.

*ED* emergency department, *IADT* International Association of Dental Traumatology.

*p-value ≤ 0.05.

## Discussion

The present study was conducted to assess the knowledge and awareness of ED physicians and residents regarding the management of DFTI. Recognizing their pivotal role as frontline responders in dental trauma cases, the study aimed to provide baseline information on the current level of knowledge among ED residents and physicians. The findings of the survey showed that the knowledge and awareness of ED residents and physicians was generally low, with no significant difference in the knowledge level among both groups (*p* > 0.05). The overall trend in responses was consistent with previous studies in identifying a gap in critical knowledge and management of DFTI amongst ED specialists [5, 16, 18–20]. One plausible reason for these findings is that the management of dental trauma cases is not generally included in medical curriculums [21].

The findings of the current study observed that the participants’ knowledge level of IADT guidelines, basic tooth anatomy, the difference between primary and permanent teeth, crown fractures, and emergency management of an avulsed tooth was insufficient. These were in contrast with a previous study by Wolfer et al., conducted on four different medical sub-specialties in ED who reported an inadequate overall knowledge of the participants. [12]. However, when knowledge and skills of the same participants were self-assessed and graded using criteria by Yigit et al., the results showed a moderate level of knowledge in managing dental trauma and avulsed teeth [6, 12]. Conversely, findings from Yigit et al. and Yeng et al. aligned with our results and highlighted the insufficient knowledge of participants in managing fractures and avulsion injuries [5, 21]. Potential explanations for these disparities include limited training opportunities or the absence of standardized protocols for teaching emergency dental care in the ED of various hospitals. Furthermore, the evolving nature of medical and dental knowledge, coupled with advancements in emergency dental care protocols following IADT guidelines, may contribute to differences in curriculum updates across institutions. This dynamic landscape poses challenges in dental education that extend beyond specific geographic or institutional boundaries.

The results of the last part of the survey in which questions were asked regarding management of maxillofacial trauma, showed that participants of our study lacked knowledge in this domain as well. However, the proportion of correct number of answers was higher compared to the second part of the questionnaire, encompassing questions on tooth anatomy and management of dental trauma. Most of the participants in our study were familiar with which radiographic investigations to be done in case of trauma to the maxillomandibular region. Despite this, there is no statistically significant difference among the groups. Our findings were in contrast with other studies that concluded that participants have more information about dental trauma than maxillofacial trauma [4, 22]. One attributable reason for this difference could be due to greater exposure of ED residents and physicians to trauma in maxillofacial region as opposed to dental trauma which may be neglected in our part of the region [23].

Regarding the factors affecting knowledge level in the management of DFTI, the present study showed that having more years of clinical experience as well as formal dental trauma training were statistically significant factors. This was in agreement with the study by Yigit et al. who also concluded that greater ED experience was a significant factor affecting knowledge level in the management of DFTI [6]. However, the result is in contradiction with earlier studies by Dawoud et al., Trivedy et al., and Ulusoy et al. concluding that greater experience had no significant impact on the knowledge level of the participants [18, 22, 24]. It is worth mentioning that majority of the contrasting literature were based on European studies [4, 12]. One attributable reason may be the introduction of “Oral and Maxillofacial Procedures” in the new European Core Curriculum for Emergency Medicine [25]. Based on this, clinical emergency medicine has been updated in the medical curriculum since 2020. However, the implications of this upgradation in curriculum remain questionable in our state [20, 26].

Regarding the participants, the present study included ED physicians and residents as the study subjects in contrast to other European & Turkish studies on the management of DFTI [4, 5, 12]. Yigit et al. included general practitioners, ED specialists, and ED residents as the study participants whereas Coskun et al. included only ED physicians and ED nurses [4, 6]. Wolfer et al. included participants who were providing emergency care in the ED, despite having basic training in various specialist areas. These medical specialties included anesthesiology, surgery, internal medicine, otolaryngology, general medicine, gynecology, and pediatrics [12]. The study participants of the present study reflect the importance of this survey since very few studies have included ED residents as the study subjects [6]. Moreover, ED residents play a crucial role as the initial responders to diverse forms of traumatic incidents, emphasizing their frontline position in the timely and appropriate management of DFTI within the ED, highlighting the comprehensive nature of their involvement.

In the present study, the questionnaire underwent validation by 6 experts of different specialties, followed by CVI calculation. Literature suggests that CVI thresholds vary depending on the number of experts involved in validation processes [27]. In our case, the contributions of all six experts resulted in a remarkably high CVI score, confirming the accuracy and robustness in efficiently capturing the needed information.

 The present study has some limitations. Firstly, there may be an inherent limitation since participation in the survey was voluntary. Higher rates of knowledge could be attributed to voluntary participation [28]. It is possible that those replying physicians have a greater interest in and awareness of oral trauma, which may have resulted in enhanced knowledge levels. Secondly, the study acknowledges the presence of reporting bias, which may result from differences in participants’ interpretations of survey questions, hesitation in responding, or susceptibility to external influences. These factors can introduce variations in the reliability and validity of the collected data [29]. Hence, it is essential to take these considerations into account when examining the potential for reporting bias in a study. Thirdly, the use of external resources such as books or media in filling out the questionnaires could introduce bias in the results. This is illustrated by a study conducted by Kooijmans et al. which reported biased outcomes when participants received assistance in completing surveys [30].

Our recommendations are future studies involving comparison groups as opposed to a single group study to evaluate the difference in the knowledge level among ED residents and physicians. Another recommendation is training sessions followed by pre & post-training assessments of ED residents and physicians regarding knowledge and management of DFTI. This will help assess the effectiveness of the training program and identify areas for improvement in the specialist’s understanding and handling of DFTI cases. Moreover, adopting a long-term approach to monitor post-training outcomes and interventions will offer valuable insights into how well the knowledge translates into practical situations. This comprehensive evaluation method will greatly enhance our strategies for handling and addressing DFTI within the healthcare system.

## Conclusions

The present study findings showed that the knowledge of ED residents and physicians who responded to the survey regarding dental trauma management is generally low. However, factors such as formal dental trauma training as well as greater years of experience are associated with the enhancement of knowledge regarding the management of these injuries. To increase the knowledge of ED specialists on DFTI, targeted training sessions, interdisciplinary seminars, case discussions, and continuing education programs should be held. These efforts may significantly improve the knowledge and awareness of ED residents and physicians related to DFTI. This improvement could positively impact the outcomes of patients seeking initial care in the ED for traumatic dentofacial injuries.

## Supplementary information

Supplementary Information 1

## Data Availability

The datasets used and/or analyzed during the current study available from the corresponding author on reasonable request.

## References

[1] Petti S, Glendor U, Andersson L. World traumatic dental injury prevalence and incidence, a meta‐analysis—One billion living people have had traumatic dental injuries. Dent Traumatol. 2018;34:71–86. 29455471 10.1111/edt.12389

[2] Çalışkan S, Delikan E, Kızılaslan S, Özbek Ö. Knowledge of Dental Avulsion Among Emergency Physicians: A Survey Study. J Pediatr Res. 2021;8:62–9.

[3] Juncar M, Tent PA, Juncar RI, Harangus A, Mircea R. An epidemiological analysis of maxillofacial fractures: a 10-year cross-sectional cohort retrospective study of 1007 patients. BMC Oral Health. 2021;21:128–38. 33731083 10.1186/s12903-021-01503-5 PMC7968332

[4] Coşkun A, Şener A, Şahin O, Ekmekcioğlu C. Knowledge and attitudes of emergency medicine physicians and nurses regarding emergency management of dentofacial trauma in pediatric patients. Arch Pediatr. 2021;28:520–4. 34507864 10.1016/j.arcped.2021.07.005

[5] Steeves ED, Tracy Mello Isabel. Perceptions of emergency department physicians regarding the management of traumatic dental injuries. Dent Traumatol. 2023;1:1–7. 10.1111/edt.12844 37039270

[6] Yigit Y, Helvacioglu-Yigit D, Kan B, Ilgen C, Yilmaz S. Dentofacial traumatic injuries: A survey of knowledge and attitudes among emergency medicine physicians in Turkey. Dent Traumatol. 2019;35:20–6. 30218627 10.1111/edt.12440

[7] Aren A, Erdem AP, Aren G, Şahin ZD, Güney Tolgay C, Çayırcı M, et al. Importance of knowledge of the management of traumatic dental injuries in emergency departments. Turk J Emerg. 2018;24:136–44. 10.5505/tjtes.2017.57384 29569685

[8] Bourguignon C, Cohenca N, Lauridsen E, Flores MT, O'Connell AC, Day PF, et al. International Association of Dental Traumatology guidelines for the management of traumatic dental injuries: 1. Fractures and luxations. Dent Traumatol. 2020;36:314–30. 32475015 10.1111/edt.12578

[9] Andreasen JO, Andreasen FM, Skeie A, Hjørting-Hansen E, Schwartz O. Effect of treatment delay upon pulp and periodontal healing of traumatic dental injuries–a review article. Dent Traumatol. 2002;18:116–28. 12110104 10.1034/j.1600-9657.2002.00079.x

[10] Boffano P, Kommers SC, Karagozoglu KH, Forouzanfar T. Aetiology of maxillofacial fractures: a review of published studies during the last 30 years. Br J Oral Maxillofac Surg. 2014;52:901–6. 25218316 10.1016/j.bjoms.2014.08.007

[11] Díaz J, Bustos L, Herrera S, Sepulveda J. Knowledge of the management of paediatric dental traumas by non‐dental professionals in emergency rooms in South Araucanía, Temuco, Chile. Dent Traumatol. 2009;25:611–9. 19843130 10.1111/j.1600-9657.2009.00835.x

[12] Wolfer S, von Hahn, Sievers D, Hohenstein C, Kauffmann P. Knowledge and skills of emergency physicians in managing traumatic dental injuries. Eur J Trauma Emerg Surg. 2022;48:2081–8. 34689226 10.1007/s00068-021-01808-8 PMC9192501

[13] Losier JH, Myslik F, Van Aarsen K, Cuddy K, Quinonez C. P085: Dental complaints in the emergency department: A national survey of Canadian EM physicians. Can J Emerg Med. 2017;19:107.

[14] Puri KS, Suresh KR, Gogtay NJ, Thatte UM. Declaration of Helsinki, 2008: implications for stakeholders in research. J Postgrad Med. 2009;55:131–4. 19550060 10.4103/0022-3859.52846

[15] WHO. World health organisation calculator. 2023. Available from: https://www.calculator.net/sample-size-calculator.html.

[16] Day PF, Flores MT, O'Connell AC, Abbott PV, Tsilingaridis G, Fouad AF, et al. International Association of Dental Traumatology guidelines for the management of traumatic dental injuries: 3. Injuries in the primary dentition. Dent Traumatol. 2020;36:343–59. 32458553 10.1111/edt.12576

[17] Fouad AF, Abbott PV, Tsilingaridis G, Cohenca N, Lauridsen E, Bourguignon C, et al. International Association of Dental Traumatology guidelines for the management of traumatic dental injuries: 2. Avulsion of permanent teeth. Dent Traumatol. 2020;36:331–42. 32460393 10.1111/edt.12573

[18] Abu-Dawoud M, Al-Enezi B, Andersson L. Knowledge of emergency management of avulsed teeth among young physicians and dentists. Dent Traumatol. 2007;23:348–55. 17991234 10.1111/j.1600-9657.2006.00477.x

[19] Al Mahmoud A, Al Halabi MH, Kowash I. Knowledge of Management of Traumatic Dental Injuries of Emergency Department Physicians and Residents in the United Arab Emirates. J Dent Child. 2019;86:24–31. 30992098

[20] Walker A, Brenchley J. It’s a knockout: survey of the management of avulsed teeth. Accid Emerg Nurs. 2000;8:66–70. 10818369 10.1054/aaen.1999.0115

[21] Yeng T, O'Sullivan AJ, Shulruf B. Medical doctors’ knowledge of dental trauma management: A review. Dent Traumatol. 2020;36:100–7. 31609070 10.1111/edt.12518

[22] Ulusoy AT, Onder H, Cetin B, Kaya S. Knowledge of medical hospital emergency physicians about the first-aid management of traumatic tooth avulsion. Int J Paediatr Dent. 2012;22:211–6. 21883562 10.1111/j.1365-263X.2011.01178.x

[23] Babar A, Saeed Z, Mahmood S. Management of traumatic injuries in the oral and maxillofacial region. Consideration during the covid pandemic. PAFMJ. 2020;70:S642–47.

[24] Trivedy C, Kodate N, Ross A, Al-Rawi H, Jaiganesh T, Harris T, Anderson JE. The attitudes and awareness of emergency department (ED) physicians towards the management of common dentofacial emergencies. Dent Traumatol. 2012;28:121–6. 22107050 10.1111/j.1600-9657.2011.01050.x

[25] European Core Curriculum for Emergency Medicine. 2017. Available from: https://eusem.org/images/pdf/European_Core_Curriculum_for_EM_Version_1.2_April_2017_final_version.pdf.

[26] Yeng T, O’Sullivan AJ, Shulruf B. Developing a prototype dental trauma e-learning course for medical education. Aust Endod J. 2022;48:44–50. 34258841 10.1111/aej.12545

[27] Yusoff MSB. ABC of content validation and content validity index calculation. Educ Med J. 2019;11:49–54.

[28] Kılınç H, Fırat M. Opinions of expert academicians on online data collection and voluntary participation in social sciences research. Educ Sci Theory Pr. 2017;17:1461–86.

[29] Institute of Medicine; Board on the Health of Select Populations; Committee on Psychological Testing, Including Validity Testing, for Social Security Administration Disability Determinations. Psychological Testing in the Service of Disability Determination. Washington (DC): National Academies Press (US); 2015. 26203491

[30] Kooijmans R, Langdon PE, Moonen X. Assisting children and youth with completing self-report instruments introduces bias: A mixed-method study that includes children and young people’s views. Psychol Methods. 2022;7:100102.

