# Supplementary material for: Knowledge and awareness of emergency department residents and physicians regarding the management of dentofacial traumatic injuries: a cross-sectional study
Source: BDJ Open. 2024 Nov 9;10:85. doi: 10.1038/s41405-024-00267-8 (PMC11550422; doi:10.1038/s41405-024-00267-8)
Supplement: Supplementary file 1 — Supplementary Information 1 [file 41405_2024_267_MOESM1_ESM.pdf]

Supplementary File 1

Questionnaire. Illustration images courtesy of iStock.com/alex-mit [4, 6, 8, 16, 17]

**Part 1:**
**1.** Gender: a) male b) female
**2.** Age (years): a) 20-25 b) 26-35 c) 36-45 d) >45
**3.** Designation: a) ED resident b) ED physicians
**4.** If resident, please specify year of residency: a) 1st b) 2nd c) 3rd d) 4th
**5.** Years of experience: a) 1-4 b) 5-10 c) 11-15 d) >15
**6.** Any presence of dentist in the family: a) Yes b) No
**7.** Have you received any trauma training in your undergraduate program: a) Yes b) No
**8.** Have you experienced dental trauma in emergency department: a) Yes b) No
**9.** If yes, how many cases of dental trauma have you encountered in ED: a) Less than 10 b) 10 – 20 c) more than 20
**10.** Are you aware of international association of dental traumatology (IADT) guidelines: a) Yes b) No
**11.** Is there a consultant dentist in your institution? a) Yes b) No

Part 2:
**1.** Can you differentiate between crown vs root fracture? a) Yes b) No
**2.** A 10-year-old boy reported with uncomplicated crown fracture of maxillary incisor teeth in ED. What is fractured teeth likely to be: a) primary teeth b) permanent teeth*
**3.** What is the initial management of uncomplicated crown fracture in the same patient mentioned in (2):   a) no treatment b) refer the patient with the tooth to the dentist   c) search for the teeth fragment*
**4.** You receive a phone call informing you that a child has been hurt and a permanent tooth has been missing. Which of the following will you recommend to parents?   a) Wrap the tooth in a clean piece of gauze or handkerchief and look for a dentist quickly   b) Replace the tooth back in the mouth as soon as possible and seek dentist*   c) Put the tooth in cold and fresh milk and look for a dentist   d) Don't know
**5.** If a patient has arrived with a knocked out (avulsed) permanent tooth in an emergency department within 15 mins after an injury, what would be the urgent management option in this case?   a) Wrap the tooth in a clean gauze and seek dentist b) Replace the tooth as soon as possible into socket and seek dentist* c) Place tooth in cold fresh milk and seek dentist
**6.** How urgent is it to replant permanent avulsed tooth?   a) within 15-30 mins* b) within 24 hours c) time is not important
**7.** Should an avulsed primary tooth be replanted?

	a) Yes b) No*
**8.**	If an avulsed tooth looks dirty, what would you do?<br>a) scrub the tooth with soap or a 70% alcohol solution disinfectant<br>b) Use an antiseptic solution, such as chlorhexidine, to clean the tooth<br>c) wash the tooth gently with water*
**9.**	What are the best storage options for an avulsed tooth:<br>a) sterile saline b) milk** c) saliva
**10.**	From which part would you hold an avulsed tooth:<br>a) crown * b) root
**11.**	A 13-year-old boy with history of trauma is brought to ED. On examination 2 upper front teeth are missing and some bleeding is visible on the gingiva. What investigations should be done in this case?<br>a) Chest radiograph*<br>b) periapical radiograph (single tooth x-ray of the site)<br>c) bronchoscopy<br>d) Don't know

Part 3:

1. Which of the following occlusions could be described as ideal jaw relationships?

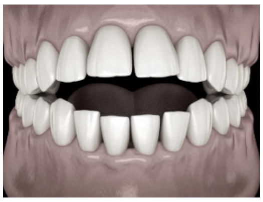

a)

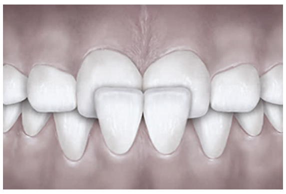

b)

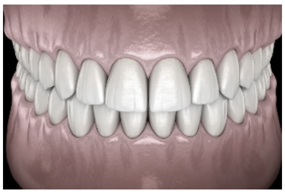

c)*

2. What is the best radiographic option in patient with simple dental trauma:

a) periapical (single tooth x-ray)*

b) orthopantomography

c) lateral cephalometric radiography

d) computed tomography

3. What is the best radiographic option in maxillofacial trauma patient:

a) periapical b) orthopantomography c) lateral cephalometric radiography

d) computed tomography*

4. In which area do you suspect a fracture in a patient who is unable to open and close their mouth due to maxillofacial trauma?

a) mandibular condyle* b) mandibular chin c) mandibular ramus
